# Mechanisms of Glucagon‐Like Peptide 1 Receptor Agonist‐Induced Facial Lipodystrophy and a Path Toward Prevention

**DOI:** 10.1111/jocd.70584

**Published:** 2025-11-28

**Authors:** Ilja L. Kruglikov

**Affiliations:** ^1^ Scientific Department Wellcomet GmbH Bruchsal Germany

**Keywords:** caveolin‐1, GLP‐1 receptor agonists, prevention

## Abstract

**Background:**

The emergence of a distinct facial appearance characterized by pronounced hollowing of the cheeks, temples, chin, and periorbital region has become a noteworthy side effect of treatment with glucagon‐like peptide 1 receptor agonists (GLP‐1RAs). This phenomenon presents a growing concern in both dermatology and aesthetic medicine. The reduction of facial adipose tissue in patients receiving GLP‐1RAs often exceeds the overall weight and fat loss typically associated with these agents, suggesting that the effect cannot be fully attributed to systemic metabolic changes alone. Instead, it raises the possibility of localized, tissue‐specific mechanisms of GLP‐1RA action within facial fat depots.

**Aim:**

In this article, we explore the underlying pathophysiology of this selective facial fat loss and discuss potential strategies for mitigating or reversing the aesthetic impact of GLP‐1RAs.

**Conclusion:**

Since the local effects of GLP‐1RAs are realized through internalization of GLP‐1 receptors (canonical pathway) or IGF‐1R (non‐canonical pathway), the suppression of mechanisms responsible for this internalization can be used to prevent the development of partial lipodystrophy after the application of GLP‐1RAs. One encouraging possibility for such a preventive intervention can be the local modulation of CAV1 in the facial adipose tissue during the GLP‐1RAs treatment course.

## 1 Introduction

Two gastrointestinal hormones, glucagon‐like peptide 1 (GLP‐1) and glucose‐dependent insulinotropic polypeptide (GIP), which exert their physiological effects through interaction with membrane receptors GLP‐1R and GIPR, correspondingly, are responsible for the postprandial glucose‐induced insulin secretion. The expression of GLP‐1 is strongly reduced in patients with type 2 diabetes, and the activation of GLP‐1 is considered a new therapeutic avenue for the treatment of this disease. The therapeutic breakthrough in this field was achieved first after the development of the GLP‐1R agonists (GLP‐1RAs), which are more stable to the enzyme dipeptidyl‐peptidase‐4 than GLP‐1. While the application of the GLP‐1RAs primarily induces the production of insulin and enhances insulin sensitivity, it is also believed to promote delayed gastric emptying, producing the sensation of satiety and decreased appetite [1]. This is the main reason why these agonists were included in weight management programs in non‐diabetic patients and why the application of drugs based on the GLP‐1RA semaglutide, became popular during the recent past.

Whereas the main known side effects after the application of the GLP‐1RAs relate to gastrointestinal disorders, quick weight loss can also cause an enhanced reduction of adipose tissue in some body areas, producing the face to look like in partial lipodystrophy. This reduction, which should be observed to some degree after all treatment methods providing rapid weight loss, has become a serious challenge for aesthetic medicine. Typical changes comprise a pronounced hollowed‐out appearance of the cheeks, temples, chin, and periorbital area caused by differential reduction of various facial fat compartments, which emphasizes the increased skin laxity and wrinkles in affected patients [2, 3, 4]. Whereas these changes are also typical for intrinsically aging skin, after rapid weight loss, they occur more quickly and at a younger age [5].

Quantitative information about facial fat reduction after prolonged exposure to GLP‐1RAs is still limited. The only known reliable assessment of facial fat loss was provided for a small group of five Ozempic patients using multiple facial imaging scans and the 3D‐Slicer program, which revealed an average reduction of the temporal fat pad and cheek fat pad of 41.8% and 69.9%, respectively [3]. This points not just to some limited reduction of fat in these compartments but reflects the induction of a partial lipodystrophy in these depots. A similar facial skin aging appearance was also observed after the application of other treatment methods associated with rapid weight loss [3]. For example, the post‐bariatric surgery reduction of the average BMI from 38 to 27 caused a volume loss in the midface and nasolabial groove regions in 86% of patients, and the perioral volume loss in 59% of patients [6].

Whilst the reduction of different facial fat compartments after the application of generalized weight loss procedures is not surprising, a quantitative difference between the reduction of the facial and non‐facial fat depots raises some questions. Application of the dual‐energy X‐ray absorptiometry to the patients on semaglutide revealed a total weight reduction of 5.9% (from 103.2 ± 4.1 to 97.1 ± 4.0 kg) and a reduction of the fat mass of 9.2% (from 43.5 ± 3.2 to 39.5 ± 3.3 kg) [7]. Thus, the total fat reduction in patients on GLP‐1RAs is significantly smaller than the shrinkage of the facial fat pads [3]. This means that facial adipose tissue reacts to rapid weight loss procedures in a different way than its abdominal counterpart. To correctly define the possible countermeasures and to choose suitable treatment options, including potentially preventive treatments to avoid the appearance of partial lipodystrophy after GLP‐1RAs, it is necessary to understand the reason for such reactions of regionally different adipose depots to the same stimuli.

## 2 Do GLP‐1RAs Directly Affect Adipose Tissue?

GLP‐1Rs and GIPRs are both expressed not just in pancreatic β‐cells, but also in different extra‐pancreatic tissues. However, it is believed that only GIPR, but not GLP‐1RAs, directly affects the adipose tissue [8]. While different studies report some direct effect of GLP‐1RAs on adipose tissue [9], there is a broad consensus in the scientific community that the weight and fat‐reducing effects of GLP‐1RAs are not caused by direct interaction of these drugs with adipocytes through the canonical pathway involving GLP‐1Rs. Indeed, according to the Human Protein Atlas, there is negligible production of GLP‐1Rs in adipose tissue at both mRNA and protein levels. On the other hand, enhanced facial fat reduction compared to general fat loss observed in patients after GLP‐1RAs therapy indicates that some direct effect of GLP‐1RAs on adipocytes in at least some fat depots might exist.

Both GLP‐1 and the GLP‐1RA liraglutide promoted differentiation of preadipocytes into adipocytes in vitro and in vivo [10]. This differentiation involved GLP‐1Rs, which were found both in undifferentiated and differentiated preadipocytes of the 3T3‐L1 cell line. The GLP‐1R knockdown induced by lentivirus containing shRNA significantly reduced adipogenic differentiation and inhibited the expression of the master regulator of adipogenesis, PPARγ, in the presence of GLP‐1 [10]. Some of these results were later confirmed in [11]. Protein expression of GLP‐1Rs during 3 T3‐L1 adipogenesis demonstrated a distinctly transitory behavior, increasing during the early stages and substantially decreasing during the late stages of differentiation [12]. Moreover, the application of liraglutide to the differentiated 3 T3‐L1 cells dramatically reduced the expression of *FASN* (key regulator of de novo lipogenesis) in a time‐ and dose‐dependent manner but did not affect the expression of *ATGL* (indicator for lipolysis). At the same time, the application of the GLP‐1R‐specific blocker exendin attenuated the reduction of *FASN*, demonstrating that this effect is directly connected with GLP‐1R activation in differentiated cells [12]. The GLP‐1R expression was found both in mature adipocytes and stromal‐vascular fraction, with substantially higher expression in the latter [13]. As demonstrated in adipose tissue biopsies obtained from obese individuals during bariatric surgery, GLP‐1Rs are much more highly expressed in visceral than in subcutaneous fat [14], suggesting that visceral adipose tissue should be more sensitive to the application of GLP‐1RAs. Randomized controlled trials with exenatide and liraglutide indeed demonstrated about 35% higher reduction of visceral compared to subcutaneous adipose tissue [15].

GLP‐1Rs were also found in epicardial adipose tissue (EAT), where their expression was higher than in subcutaneous white adipose tissue (sWAT) of the same individuals [16]. Remarkably, diabetic and obese patients on the GLP‐1RA liraglutide demonstrated a substantial (about 36%) reduction in the EAT thickness [17]. Like the fat reduction described in facial compartments after the application of GLP‐1RAs [3], the shrinkage effect of liraglutide on EAT was significantly higher than the total weight or fat tissue reduction in these patients, which was difficult to explain with just a systemic effect of liraglutide.

Importantly, EAT and buccal fat compartment share some similarities. EAT is a local visceral fat depot located between the visceral pericardium and myocardium. On the other hand, the cross‐sectional area of the buccal compartment measured with the use of computer tomography demonstrated a significant linear correlation with the cross‐sectional area of intra‐abdominal visceral fat, but no correlation with subcutaneous fat or BMI in investigated individuals [18]. Buccal fat can influence the appearance of cheek and temporal fat pads through its body and temporal extension parts, respectively. Visceral fat contains larger adipocytes and is more insulin resistant compared to sWAT. This contradicts a preferential reduction of this fat through GLP‐1RAs, which, according to the existing paradigm, should primarily induce the production of insulin and enhance insulin sensitivity. This paradox can be explained by a higher expression of GLP‐1Rs in visceral adipose tissue, as observed in [14]. Whereas the effect of GLP‐1RAs on buccal fat was not investigated in detail, it is reasonable to assume that this fat pad, demonstrating a high correlation with intra‐abdominal visceral fat, should have an increased expression of GLP‐1RAs.

Taken together, GLP‐1Rs are expressed in adipocytes at low levels, and this expression is dependent on the degree of adipocyte differentiation, the type of adipose tissue, and the metabolic conditions in this tissue. However, it is not clear whether this canonical activation of the GLP‐1Rs is the main pathway for the enhanced fat reduction observed in facial fat depots and in EAT after application of GLP‐1Ras, or if a non‐canonical effect through activation of other receptors is also involved.

A non‐canonical effect of GLP‐1 can be realized through stimulation of insulin‐like growth factor 1 receptor (IGF‐1R). GLP‐1 robustly stimulated IGF‐1R's expression in a time‐dependent manner both in MIN6 (mouse pancreatic β‐cell line) and in primary β‐cells, activated IGF‐1/IGF‐2 autocrine loop and Akt phosphorylation, whereas reduction of IGF‐1R expression suppressed GLP‐1‐induced protection of β‐cells against apoptosis [19]. Whereas human cardiac fibroblasts bear no GLP‐1Rs, they display an upregulation of elastin fiber production after culturing with GLP‐1, which is triggered by activation of IGF‐1Rs [20].

## 3 The Role of Superficial and Deep Adipose Tissue in Facial Lipodystrophy After Application of GLP‐1RAs

Every facial fat compartment can be subdivided into superficial and deep fat layers [21, 22]. The superficial fat layer, also known as dermal WAT (dWAT) in rodents [23] and sometimes named skin‐associated adipose tissue (SAAT) in humans [24], consists of a special type of labile adipocytes, which can undergo different phenotypical transformations even under physiological conditions [24, 25]. This layer demonstrates sexual dimorphism and expands with progressive aging [26], whereas in advanced aging it is reduced in the same way as other fat depots. Such temporal behavior of dWAT was recently confirmed in cadavers using B‐mode ultrasound with a frequency of 24 MHz together with sonoelastography [27]. Adipocytes from this superficial layer can locally react to internal and external factors with a quick modification in their number and cell volumes demonstrating either expansion or reduction of the dWAT layer. Quick and substantial expansion of dWAT occurs, for example, during reactive adipogenesis—differentiation of dermal preadipocytes into mature adipocytes accompanied by expression of antimicrobial peptides in response to invading pathogens [28].

As it was recently demonstrated in a murine model using the triglyceride radiotracer triolein, skin is the main target for deposition of dietary triglycerides, which accumulate in the epidermis and dWAT for weeks after feeding [29]. This makes dWAT an important depot for skin lipids that can slowly exchange them with other skin structures. Correspondingly, a 30% caloric restriction for 3 weeks dramatically reduced both the thickness of the epidermis and the dWAT layer [29], which means that dWAT can quickly and effectively react to both caloric gain and restriction. A significant caloric reduction occurs in all rapid weight loss procedures, including exposure to GLP‐1RAs, and it should have a similar effect on dWAT in humans. From here, the superficial facial fat layers must demonstrate a more rapid reduction in response to the quick weight loss procedures than the corresponding deep fat layers, which was indeed described in [3]. It should be noted that this effect exists just during caloric restriction, and a subsequent exposure to a high fat diet should restore the content of lipids and the structure of dWAT, making this modification at least partly reversible.

The deeper facial fat layers, including sWAT and buccal fat, demonstrate a reduction in their thicknesses and ample modification of their intracellular matrices both in advanced aging and during rapid weight loss. Laxity of the skin is determined by the mechanical behavior of the composite skin/sWAT, and modification of the local mechanical properties of the composite skin/WAT after rapid weight loss reduces skin resistance to mechanical deformation [30]. The Young's modulus (measure of material stiffness) of this composite is mainly determined by the mechanical properties of the skin under mechanical loading parallel to the skin surface, and by sWAT properties under mechanical loading perpendicular to the skin [31]. Adipose tissue contains two types of collagen networks—inter‐ and pericellular fibrotic structures, whereas the mechanical properties of sWAT are mainly determined by pericellular fibrotic structures consisting of collagen types IV and VI [31]. The Young's modulus of sWAT linearly increases with the thickness of the pericellular fibrosis around adipocytes and decreases with progressive hypertrophy of adipocytes. Thus, a reduction in pericellular fibrosis caused by rapid weight loss makes the composite skin/sWAT much softer, thereby promoting the appearance of laxity [31]. Correspondingly, it is reasonable to suppose that during rapid weight loss, facial fat volume decreases first through modification of the dWAT/SAAT and then through a reduction of the sWAT/buccal compartments. This could be an important reason for the development of apparent aging signs among these individuals and should influence the choice of skin rejuvenation treatment strategy for these patients.

## 4 Do GLP‐1RAs Directly Affect the Skin?

It is well‐established that GLP‐1RAs can induce both adverse and beneficial cutaneous effects [32, 33]. One important adverse effect, which was never discussed in the literature before, is the rapid reduction of the lipid content in the skin through caloric restriction [29]. This reduction in epidermis and sebum can be at least partly compensated for by the topical application of oily products. A comprehensive analysis of the skin structure after rapid weight loss was recently given in [34]. Patients after rapid weight loss demonstrated a reduction of the thick and an increase in the thin collagen fibers, indicating a change in collagen balance. The elastic fiber density significantly increases in the skin of these patients, providing its higher elasticity, compensating for the reduction of the underlying fat layer [34, 35].

Among beneficial outcomes, improvement of *psoriasis* and *hidradenitis suppurativa*, accelerated healing of surgical wounds, and induced closure of chronic wounds, as well as possible anti‐aging effects, were reported. Recent systematic reviews for GLP‐1RAs in the treatment of *psoriasis* [36] and *hidradenitis suppurativa* [37] concluded that GLP‐1RAs demonstrate beneficial effects on these inflammatory skin conditions, but the mechanism of their action is still not fully understood. Incretin‐based therapy was associated with accelerated healing of surgical wounds in rats, whereas such therapy in humans demonstrated a lower risk of diabetic foot ulcers [38]. The positive effect of GLP‐1RAs in anti‐aging is more theoretical and based on their reported ability to protect against oxidative stress, cellular senescence, and chronic inflammation [39]. These results clearly demonstrate that the effect of GLP‐1RAs is much more comprehensive than a mechanism solely based on enhanced insulin secretion or a decrease in appetite in patients undergoing treatment.

The main problem with the interpretation of these results relates to contradictory reports concerning the production of GLP‐1Rs in the skin, which, like in adipose tissue, shifts the mechanism of action from canonical to non‐canonical pathways. Increased expression of GLP‐1Rs was found in psoriatic plaques; however, human keratinocytes in this study demonstrated no expression of GLP‐1Rs. Higher expression of GLP‐1Rs was mainly connected to infiltration of these plaques with immune cells [40]. As we mentioned above, human cardiac fibroblasts cultured with GLP‐1 demonstrate an enhanced production of elastin fibers, and this effect is triggered through activation of the non‐canonical IGF‐1R pathway [20].

The pathophysiology of *psoriasis* and *hidradenitis suppurativa* (as well as of some other inflammatory skin conditions), along with acute and chronic wounds and skin aging, was recently linked to the signaling protein caveolin‐1 (CAV1)–the main structural component of caveolae, which are plasma membrane invaginations forming nanodomains with a typical size of 50–100 nm. Caveolae are engaged in rapid adaptation to cellular volume change, in various signal transduction processes, and in the processes of endo‐ and exocytosis. The CAV1 content is very low in inflammatory psoriatic skin [41], and this reduction is a clear pathophysiological factor in different types of psoriasis, such as plaque, pustular, palmoplantar, and nail psoriasis, but not in psoriasis guttate or inverse [42]. Low expression of CAV1 is also typical in fibrotic diseases, hypertrophic scars and keloids [43] as well as in acute wounds. Contrary to this, in chronic wounds, such as diabetic foot ulcers or *ulcus cruris venosum*, its expression is very high, especially near the edges of the wound [44, 45]. This prevents the penetration of keratinocytes into the wound and blocks the healing process. In accordance with this, the application of the antidiabetic drug mevastatin, which causes a reduction of CAV1, provided significant improvement in the healing of chronic wounds [46]. Similarly, high expression of CAV1 is typical in *h* *idradenitis suppurativa* [47]. CAV1 is also in a major way involved in skin aging and can serve as a target for anti‐aging therapy [26].

Altogether, CAV1 is currently considered not just an important pathophysiological factor in different skin conditions, but also an effective target for their treatment [48, 49]. Thus, the interaction between CAV1 and canonical (through GLP‐1R) or non‐canonical (through IGF‐1R) pathways should be involved in the beneficial effects of GLP‐1RAs on the skin.

## 5 What Is the Role of CAV1 on the Impact of GLP‐1RAs?

Like many other signaling receptors, GLP‐1Rs are co‐localized and mechanistically interact with CAV1 in caveolae. Binding of agonists to human GLP‐1Rs activates intracellular signaling pathways through internalization of this receptor via clathrin‐ or CAV1/DYN2‐dependent endocytosis [50, 51, 52, 53]. This raises the question of whether long‐lasting partial facial lipodystrophy after application of GLP‐1RAs or in a similar way after other rapid weight loss procedures can be caused by a mechanism connected with this endocytosis.

WAT is a reservoir for various pathogens and their products, such as lipopolysaccharides (LPS), and it is equipped with defense mechanisms connected with the activation of innate immunity [54]. Remarkably, adipocytes express a number of different complement factors even in the absence of opportunistic pathogens. Pathological changes involving WAT demonstrate substantial, although distinct, dysregulation of the complement pathway in obesity, lipedema, or lipodystrophy [54].

Generally, obesity and type 2 diabetes are associated with what is referred to as “metabolic endotoxemia”. This involves the translocation of LPS from the intestine into circulation caused by decreased barrier function of the intestine (“leaky gut” syndrome) and is characterized by increased accumulation of pathogens and LPS, causing a low‐grade inflammation in various fat depots. Thus, it is highly likely that patients applying GLP‐1RAs for weight control have some degree of leaky gut. Of note, increased permeability of the intestine was connected to increased accumulation of ethanolamines in the gut causing a weakening of the tight junction complexes in the intestinal wall [55].

Whereas it is generally believed that GLP‐1RAs improve gut permeability and thus reduce endotoxemia, currently, there is no reliable data supporting this model [56]. Application of linagliptin (a DPP‐4 inhibitor) significantly reduced inflammation in visceral and subcutaneous adipose tissues as well as ameliorated the hypertrophy of adipocytes [57]. On the other hand, gastroduodenal and small intestinal permeabilities decreased, whereas colonic permeability increased after laparoscopic sleeve gastrectomy [58]. Thus, the application of rapid weight loss procedures does not generally improve intestinal permeability, as proposed by different authors, and can theoretically even lead to an opposite effect.

Complement factor D (CFD) is a key and rate‐limiting factor in the activation of the alternative complement pathway. CFD catalyzes the formation of C3 convertase, which is important for the development of complement‐dependent cytotoxicity through the production of the membrane attack complexes (MACs, or C5b‐9), and its expression is increased in serum and adipose tissue of patients with obesity [59]. Remarkably, CFD expression was also found to be significantly increased in serum and adipose tissue of patients with Barraquer–Simons syndrome (an acquired partial lipodystrophy with a cephalocaudal loss of subcutaneous adipose tissue) [60]. As we argued recently, WAT is constitutively equipped with many of the components of the complement fixation pathway, which allows its quick reaction against pathogens and is related to a risk of production of MACs, which can cause both lysis of pathogens and autolysis of host adipocytes, contributing to the regulation of the cellularity in WAT and development of opposite WAT conditions, such as lipedema and lipodystrophy [54, 61].

To avoid the complement‐induced autolysis, adipocytes have several defense mechanisms, among others, those connected with the expression of inhibitors that block pore assembly in the plasma membrane (e.g., CD59), producing shedding of the opsonins from the plasma membranes (by the matrix metalloproteinase 14, MMP14) or providing internalization of MACs through the mechanism of CAV1/DIN2‐dependent endocytosis [54, 61]. These processes enable a broad range of physiological and pathological conditions, from WAT hypertrophy to lipodystrophy, and include the regulation of insulin resistance in adipocytes, since insulin receptors in the plasma membranes are also localized to caveolae and thus must be inevitably utilized by enhanced CAV1‐dependent endocytosis [54].

Hypertrophic WAT demonstrates a substantial overexpression of all components of the axis CD59/MMP14/CAV1 compared to physiologically normal WAT, which provides a stronger protection of adipocytes from autolysis. On the other hand, suppression of these pathways through reduction or knockdown of some components of the CD59/MMP14/CAV1 axis can lead to enhanced autolysis of adipocytes and is typical in a local lipodystrophy [54].

It is known that CD59 can be easily inactivated through its glycation at lysine 41, which normally happens in hyperglycemic states [54]. On the other hand, the activation of GLP‐1 (and thus also of GLP‐1RAs) reduces MMP14 and endoglin, which is part of the TGF‐β receptor complex [62]. Consequently, the main defense mechanism against MACs in obese individuals with hyperglycemia should be CAV1/DYN2‐dependent endocytosis. Of note, CAV1 level is downregulated in rapidly dividing cells and dramatically upregulated at confluency [63]. Additionally, IGF‐1R and CAV1 are co‐localized, and CAV1 is essential for internalization of IGF‐1R [64].

Altogether, CAV1‐dependent endocytosis activated by the complement cascade can promote the effect of GLP‐1RAs on both adipocytes and spatially adjacent skin cells through enhanced internalization of IGF‐1Rs and GLP‐1Rs and thus be involved in local facial fat reduction. Future research will be needed to investigate this mechanism in detail.

## 6 Is CAV1 a Possible Target for Prevention?

Different non‐invasive aesthetic procedures were proposed to reduce facial lipodystrophy after rapid weight loss, among others, application of dermal fillers, radiofrequency (RF) microneedling, and some energy‐based devices [5]. The effect of dermal fillers is temporally restricted and demands multiple injections in different fat compartments. Application of RF microneedling is believed to stimulate the production of new collagen and elastin, thus enhancing skin rejuvenation. However, the tightening effect of RF microneedling on the skin is normally caused by local skin damage followed by tissue regeneration and the development of fibrosis in the vicinity of the thermocoagulated areas. Different energy‐based devices, including lasers or High‐Intensity Focused Ultrasound (HiFU), were proposed for the treatment of facial side effects after application of GLP‐1RAs because of their ability to produce some skin tightening effect. Additionally, it was proposed to include in these procedures autologous fat transfer to increase the concentration of adipose‐derived stem cells as well as composite fat grafting combining autologous fat with stromal‐vascular fraction [65]. These claims, however, remain controversial and must be clinically approved in future research. Contrary to this, a physiological and more comprehensive treatment avenue for improvement of these side effects can be based on the modulation of CAV1 content in the target tissue. Local reduction of CAV1 content in facial fat depots during generalized application of GLP‐1RAs or other rapid weight loss procedures reduces the CAV1/DIN2‐dependent endocytosis in this area and thus decreases the probability of GLP‐1RA‐induced facial lipodystrophy.

There are several therapeutic approaches to modulate the CAV1 content, which can be subdivided into three classes: approaches based on the application of drugs, hyperthermia, and very high frequency ultrasound [43]. Caveolae are linked to the actin cytoskeleton, and the application of a transient mechanical stretch to the cells can cause either stiffening or fluidization of the cytoskeletal structures. This effect is strongly dependent on the mechanical strain (relative deformation) and the strain rate [43]. In contrast, the application of higher ultrasound intensity and higher ultrasound frequencies induces higher mechanical stress in cells, causing stronger fluidization of the cytoskeletal structure and thus can more effectively reduce CAV1 content in the target area [66]. This effect can be additionally enhanced by the quasi‐simultaneous application of ultrasound waves of different frequencies, known as LDM waves [67, 68], which indeed demonstrated a strong and long‐lasting skin tightening effect as confirmed by sonoelastography [67].

## 7 Conclusions

Partial facial lipodystrophy induced by the exposure to GLP‐1RAs is a serious challenge for aesthetic medicine. Considering the rapidly growing number of patients receiving GLP‐1RA therapy, reliable treatment methods that do not exert a serious impact on skin longevity are needed to prevent or manage this side effect. Since the local effects of GLP‐1RAs are realized through internalization of GLP‐1 receptors (canonical pathway) or IGF‐1R (non‐canonical pathway), the suppression of mechanisms responsible for this internalization can be used to prevent the development of partial lipodystrophy after application of GLP‐1RAs. One encouraging possibility for such a preventive intervention can be the local modulation of CAV1 in the facial adipose tissue during the GLP‐1RAs treatment course. This can be achieved with a few different mechanisms, including the use of very high‐frequency ultrasound waves.

## Funding

The author has nothing to report.

## Ethics Statement

The author has nothing to report.

## Consent

The author has nothing to report.

## Conflicts of Interest

I.L.K. is the managing partner of Wellcomet GmbH. Wellcomet GmbH provided support in the form of salaries for I.L.K. but did not have any additional role in the decision to publish or in the preparation of the manuscript. The commercial affiliation of I.L.K. with Wellcomet GmbH does not alter the adherence to all journal policies on sharing data and materials.

## Data Availability

Data sharing not applicable to this article as no datasets were generated or analyzed during the current study.
